# On Petit’s Operation (the Operation without Opening the Sac) for Strangulated Hernia

**Published:** 1848-09

**Authors:** 


*On Petit's operation (the operation without opening the sac) for Strangulated Hernia.* By JAMES LUKE.

The object of the author in this commuication was to place before the profession the result of his experience in operating for strangulated hernia, without opening the sac. He remarked, that though experience was the only fair test by which the relative merits of this and the ordinary operation could be decided, the subject was encompassed by many obstacles, such as the impossibility of obtaining exactly parallel cases, the importance of not mixing the observations of different surgeons, or judging from selected cases. To obviate these difficulties and sources of fallacy, the author has yielded up the whole of his experience on the subject of Petit's operation, which is the only mode of operating he has adopted since the year 1841, as an ordinary practice. Being unable to supply from his own case-book the result of cases operated on by opening the sac, the author appealed to the experience of others, referring especially to statistical details given by M. Texter, Mr. South, M. Malgaigne, and collected at the London Hospital, and from the British journals generally, which gave a return of mortality of from one-third to more than one-half. Where the taxis is successful similar statistics prove that a fatal result is very rare. The conclusion which seems naturally to flow from these facts is, that operative interference should be deferred, and the taxis pursued as long as it offers any prospect of a successful issue—an inference, however, which the author considers to be fallacious and mischievous in its tendency, as involving rife source of procrastination, which in itself is the too frequent cause of non-success, attending the operation. This assertion is borne out by statistical details of cases operated on at different periods, after the establishment of symptoms of strangulation. The author then proceeded to remark that the desideratum appeared to be, the introduction of an operation by which the taxis would be aided, but without incurring the risk attending the ordinary operation, by exposure of the contents of the hernial sac; and these objects he considered to be fulfilled by Petit's operation. Inclusive of selected cases occurring between 1831 and 1841, the author stated that he had attempted the performance of Petit's operation in eighty-two instances, which, with four exceptions, likewise comprised all the cases that had come under his care since 1841. Of this number the operation was completed, without opening the sac, in fifty-seven. In twenty-five it was necessary to open the sac to complete a reduction of the hernial contents—the opening varying in extent from half an inch to one inch and a quarter. With respect to the mortality amongst these patients,—of the fifty-seven in whom the sac remained unopened, seven died; of the twenty-five in whom the sac was opened, eight died. The author considered, however, that for statistical purposes, it was preferrable to exclude the selected cases, (twenty-six in number,) together with four other patients, of whom three were considered moribund at the time the operation was performed, and the fourth died from secondary stricture, six weeks afterwards. Of the remaining fifty-two patients, the sac was opened in twenty-one, of which three died, and not opened in thirty-one, of which two died. Of the cases in which the sac was opened, in ten, strangulation of the contents had existed, before the operation was performed, under twenty-four hours, of which number one died; in eight, above forty-eight hours, of which one died. Of the unopened cases, the strangulation had existed in thirteen under twenty-four hours, of which not one died; in eleven, under forty-six hours, of which one died. The author considered it important that the small size of the opening made into the sac, in the former class of cases, should be borne in mind, as it doubtless had an important influence in diminishing the ratio of mortality attached to this mode of operating. He then passed on to further details relating to the above cases and the reason for opening the sac, and stated that of the fifty-two instances cited, twenty-nine were fermoral, twenty inguinal, and three were umbilical hernia. He further pointed out the conclusion, from an analysis of the foregoing cases, that Petit's operation has proved most successful in the femoral form of hernia. In cases of inguinal hernia, the author limits his incision to a longitudinal division of the skin and fascia over the neck of the sack, of which cut the seat of stricture should be the centre. He then partially incises or scarifies the neck of the sac, (if the seat of stricture, as it usually is,) so as only partially to divide it, and so that it shall yield to the subsequent application of the taxis. In femoral hernia, he considers it very desirable to avoid, as much as possible, interfering with the tumor in conducting the operation, and therefore recommends that a similar proceeding should be adopted—the centre of the perpendicular incision in this case being between the upper part of the tumour and the abdominal surface. Poupart's ligament is thus reached by carrying the finger from above downwards, and the stricture is divided on a director, introduced into the femoral ring. The operation advocated by the author is not considered by him to be applicable in umbilical hernia, except where it is of small dimensions. The author concluded by noticing and combatting the various objections which have been raised to Petit's operation, and by insisting on its value, apart from other considerations, as holding out inducement to surgeons to proceed with less delay to the performance of the operation, as he considered that procrastination, arising from the dread of having recourse to the more severe operative interference ordinarily adopted, was in itself, (as already remarked) a rife cause of the mortality which unhappily has too generally attended these cases.—*London Lancet.*

